# Comparison of General and Liver-Specific Prognostic Scores in Their Ability to Predict Mortality in Cirrhotic Patients Admitted to the Intensive Care Unit

**DOI:** 10.1155/2021/9953106

**Published:** 2021-09-24

**Authors:** Pedro Paulo Costa e Silva, Liana Codes, Fernanda Ferreira Rios, Carolina Pedreira Esteve, Murilo Tavares Valverde Filho, Douglas Oliveira Carmo Lima, Geraldo Fernandes de Almeida Filho, Maria Clara Alves Morais, Bruno Calazans Lima, Paulo Bravo de Oliveira Chagas, Ney Boa-Sorte, Paulo Lisboa Bittencourt

**Affiliations:** ^1^Escola Bahiana de Medicina e Saúde Pública (EBMSP), Salvador, Brazil; ^2^Gastroenterology and Hepatology Unit, Hospital Português (HP), Salvador, Brazil

## Abstract

**Introduction:**

Acute Physiology and Chronic Health Evaluation (APACHE) II and III and Sequential Organ Failure Assessment (SOFA) are prognostic scores commonly used in the intensive care unit (ICU). Their accuracy in predicting mortality has not been adequately evaluated in comparison to prognostic scores commonly used in critically ill cirrhotic patients with acute decompensation (AD) or acute-on-chronic liver failure (ACLF).

**Aims:**

This study was conducted to evaluate the performance of prognostic scores, including APACHE II, SOFA, Chronic Liver Failure Consortium (CLIF-C) SOFA, Child–Turcotte–Pugh (CPS), Model for End-Stage Liver Disease (MELD), MELD-Na, MELD to serum sodium ratio (MESO) index, CLIF-C organ failure (CLIF-C OF), CLIF-C ACLF, and CLIF-C AD scores, in predicting mortality of cirrhotic patients admitted to the ICU. *Patients and Methods*. A total of 382 patients (280 males, mean age 67.3 ± 10.6 years) with cirrhosis were retrospectively evaluated. All prognostic scores were calculated in the first 24 hours of ICU admission. Their ability to predict mortality was measured using the analysis of the area under the receiver operating characteristic curve (AUC).

**Results:**

Mortality was observed in 31% of the patients. Analysis of AUC revealed that CLIF-C OF (0.807) and CLIF-SOFA (0.776) had the best ability to predict mortality in all patients, but CLIF-C OF (0.749) had higher prognostic accuracy in patients with ACLF. CLIF-SOFA, SOFA, and CLIF-C AD had the highest AUC values in patients with AD, with no statistical difference (*p*=0.971).

**Conclusions:**

When compared to other general or liver-specific prognostic scores, CLIF-C OF, CLIF-SOFA, SOFA, and CLIF-C AD have good accuracy to predict mortality in critically ill patients with cirrhosis and patients with AD. According to the clinical scenario, different scores should be used to provide prognosis to patients with cirrhosis in the ICU.

## 1. Introduction

End-stage liver disease, particularly due to hepatitis B and C, alcoholic liver disease, and nonalcoholic steatohepatitis, accounts roughly for 1.16 million deaths worldwide [1]. It usually evolves over several years from compensated to decompensated cirrhosis, which is identified by the onset of acute decompensation (AD) with the development of hepatic encephalopathy (HE), ascites, and variceal hemorrhage (VH) [2, 3]. Further decompensation usually is preceded by recurrent ascites, HE, VH, and persistent jaundice, leading frequently to the terminal stage of cirrhosis characterized by occurrence of acute kidney injury (AKI), hepatorenal failure, and acute-on-chronic liver failure (ACLF), usually triggered by bacterial infections [2–5]. Patients with either AD or ACLF usually require admission to the intensive care unit (ICU) for monitoring organ dysfunction or organ support [6]. In those individuals, in-hospital mortality was shown to vary from 39% to 83%, depending on the reason for ICU admission, presence of organ failure, and sepsis [7].When compared to patients without cirrhosis, critically ill patients with the disease have more infections at ICU admission, increased overall mortality, and increased mortality due to sepsis or septic shock [8].

Several ICU and liver-specific scores have been used to predict outcomes of critically ill patients with cirrhosis [7, 9–14], as well as futility rules to withhold intensive care support [15, 16]. The most often used ICU scores are Acute Physiology and Chronic Health Evaluation (APACHE) II and III and Sequential Organ Failure Assessment (SOFA) scores [17], whereas liver-specific scores routinely applied to patients with cirrhosis are Child–Turcotte–Pugh (CTP) and Model for End-Stage Liver Disease (MELD) scores [18, 19]. Both were designed to predict mortality in patients with cirrhosis, respectively, after surgery [18] and transjugular intrahepatic portosystemic shunt (TIPS) placement [19]. While CTP is commonly used in clinical practice to assess disease severity, MELD is also currently used for indication and prioritization for liver transplantation [20].

Recently, MELD has been updated to incorporate serum sodium (sodium MELD (MELD-Na)) and MELD to serum sodium ratio index (MESO index) [21–23], age, and serum sodium (integrated-MELD (iMELD)) [24], attempting to improve prognostication.

Due to its better assessment of organ failure, ICU scores usually have a better accuracy to predict mortality, when compared to CTP and MELD scores [12–14]. Recently, the concept of ACLF was introduced in the literature by the Chronic Liver Failure Consortium (CLIF-C) to describe a syndrome characterized by advanced chronic liver disease associated with organ failure and a 28-day mortality higher than 15% [25, 26]. The authors have employed a prospectively validated modified SOFA score (CLIF-SOFA) to characterize CLIF-C OF and proposed two new prognostic scores: CLIF-C ACLF [27] for patients with ACLF and CLIF-C AD [28] for patients with AD of cirrhosis, without ACLF.

It is important to note that the CLIF-C criteria for ACLF have not been endorsed worldwide, and different definitions have been proposed by the Asian Pacific Association for the Study of the Liver-ACLF Research Consortium and the North American Consortium for the Study of End-Stage Liver Disease (NACSELD) [29].

The purpose of the present study was to evaluate the accuracy of liver-specific prognostic scores, such as CTP, MELD, and its variants MELD-Na, iMELD, and MESO index, as well as ICU scores, such as APACHE II and SOFA, in their ability to predict in-hospital mortality of cirrhotic patients admitted to the ICU with either AD of cirrhosis or ACLF and also to assess the performance of CLIF-C AD and CLIF-C ACLF, respectively, in those patients with either AD or ACLF.

## 2. Patients and Methods

All patients admitted to the Gastroenterology and Hepatology Unit of Hospital Português, from January 2012 to June 2018, with AD of cirrhosis or ACLF, were retrospectively reviewed. This ICU is a referral unit for critically ill patients with cirrhosis in Salvador, Brazil. The diagnosis of cirrhosis was based on clinical, biochemical, and echographic findings, as well as liver histology, whenever liver biopsy results were available. The etiology of cirrhosis and clinical features responsible for ICU admission were recorded in all patients. All cirrhotic patients admitted in the postoperative period of abdominal surgery, including liver transplantation, intra-arterial chemoembolization for hepatocellular carcinoma, and patients with HIV coinfection or advanced liver cancer were excluded from the study. Data regarding demographics, presence of comorbidities, cause of cirrhosis, clinical features, and baseline laboratory parameters including leucocyte counts, platelets, international normalized ratio (INR), total bilirubin, serum sodium, albumin, and creatinine levels were collected. General ICU and liver-specific prognostic scores were calculated within 24 hours of ICU admission, including updated Charlson Comorbidity Index (CCI), SOFA, APACHE II, CTP, MELD, MELD-Na, MESO-index, and iMELD, as previously described [17–19, 21, 23, 24, 30]. Patients were categorized into two groups, according to the presence of ACLF or AD without ACLF [31]. In addition, CLIF-C ACLF [27] and CLIF-C AD [29] scores were calculated on day one of ICU admission in patients, respectively, with ACLF and AD of cirrhosis. Acute kidney injury was diagnosed according to International Club of Ascites definition [32]. ACLF and AD of cirrhosis were evaluated in the first 24 hours of admission and graded based on CLIF-C criteria [26, 31]. NACSELD definition of ACLF [33] was also used based on parameters obtained within the first 24 hours in the ICU for better characterization of the patients. The presence of organ failures was assessed based on definitions of either CLIF-C or NACSELD [26, 27, 31, 33]. Patients were followed up until death, liver transplantation, and 28-day survival. The primary outcome was in-hospital mortality or transplant-free survival. This study was approved by the Ethics Committee in Research of Hospital Português, Salvador, Bahia.

### 2.1. Statistical Analysis

Dichotomous variables are presented in text and tables as numbers and percentage and continuous variables were expressed as mean ± standard deviation (SD) or as median and interquartile range, respectively, whether the distribution was normal or skewed. Demographic, clinical, and laboratorial variables were comparing between survivors and nonsurvivors using the chi-square test or Fisher's test for categorical variables or Student's *t*-test or the Mann–Whitney *U* test for continuous variables when appropriate. All scores were compared using nonparametric receiver operator characteristic (ROC) curves with respective 95% confidential interval (95% CI). The areas under the curve (AUC) provided the discriminative ability of the score and were compared as previously described [34]. Additionally, the prognostic score with the highest AUC obtained was considered a gold standard ROC curve. The other scores were compared to the gold standard using the Bonferroni-adjusted significance probability. In this analysis, models with an AUC equal to or greater than 0.7 were considered clinically significant. The Youden index was used to identify the optimal cut-off point for each score [35], and the corresponding sensitivity, specificity, positive, and negative predictive value (PPV and NPV) with respective 95% CI and likelihood ratio positive (LR+) and negative (LR−) were calculated. Statistical analyses were performed with the Statistical Package for Social Sciences (SPSS Inc., Chicago, IL, USA), version 21.0 for Windows, Stata for Mac (Stata Corp LLC., Texas, TX, USA), version 13.0, and OpenEpi, version 3.01 [36]. A *p* value <0.05 was considered significant. Quintile's cut-off points to Apache II, MELD-Na, SOFA, CLIC-SOFA, CLIC-C OF (for all patients), CLIF-C ACLF (for patients with ACLF), and CLIF-C AD (for patients with cirrhosis with AD), in addition to sensitivity and specificity parameters, were obtained using TG-ROC curves with graphic analysis, using Prism for Mac, version 9.1.2 (GraphPad Software, San Diego, California USA).

## 3. Results

A total of 382 consecutive patients (280 males, mean age 67.3 ± 10.7 years) were admitted to the ICU due to AD of cirrhosis (*n* = 204) or ACLF (*n* = 178).  Table 1 shows demographics and baseline clinical and laboratory features of these patients. Most of them had alcoholic liver disease (29%), cryptogenic cirrhosis (25%), hepatitis C (19%), or nonalcoholic steatohepatitis (11%). The main clinical features of those individuals were bacterial infections with or without sepsis or septic shock (*n* = 233), HE (*n* = 211), AKI (*n* = 123), and VH (*n* = 24). Among the patients, 321 had concurrent ascites (84%). According to CLIF-C criteria [26, 27, 31], 178 patients (47%) had ACLF grade I (*n* = 90), grade II (*n* = 36), or grade III (*n* = 52). The remaining patients (53%) did not fulfill the proposed CLIF-C criteria for ACLF and were categorized as AD of cirrhosis. Using NACSELD definition (33), only 33 patients (9%) had ACLF. Mechanical ventilation and vasopressors were required at ICU admission, respectively, in 7% and 5% of the cases. Most patients had advanced cirrhosis with several comorbidities presenting, respectively, mean CCI, APACHE II, CTP, and MELD scores of 7 ± 3, 15 ± 6, 10 ± 2, and 18 ± 8. Other ICU and liver-specific prognostic scores, calculated on day one, are shown in Table 1. Mortality was observed in 118 patients (31%), mainly due to septic shock (*n* = 83), ACLF (*n* = 12), hypovolemic shock (*n* = 4), respiratory failure (*n* = 4), AKI (*n* = 3), or other causes (*n* = 12). Mean (SD) ICU and length of hospital stay were 6 ± 6 and 15 ± 12 days, respectively. Demographics, clinical features, and outcomes of patients with ACLF and those with AD of cirrhosis are shown separately in Table 1. In this cohort, only 6.5% of patients underwent liver transplantation. A total of 249 (65%) patients had 28-day transplant-free survival.

**Figure 1:**
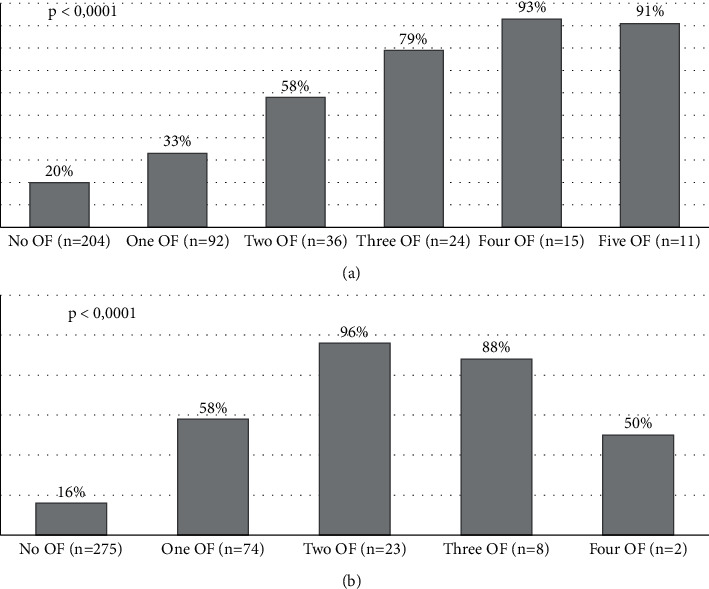
Mortality according to the number of organ failures defined by (a) CLIF-C and (b) NACSELD criteria. OF, organ failure; CLIF-C, Chronic Liver Failure Consortium; NACSELD, North American Consortium for the Study of End-Stage Liver Disease.

Mortality rate was significantly associated with clinical features of bacterial infections, HE, and AKI (Table 2). Patients with ACLF assessed by either CLIF-C or NACSELD criteria had higher mortality when compared to their counterparts without ACLF (Table 2). As expected, increased risk of death was associated with the number of organ failures, assessed by either CLIF-C or NACSELD criteria (Figure 1). Other variables associated with in-hospital mortality were creatinine and bilirubin levels, INR, and leukocyte count, as well as the need for vasopressors and mechanical ventilation at admission. All general and liver-specific prognostic scores were significantly higher in nonsurvivors when compared to their counterparts who were discharged alive from the hospital, except for the CLIF-C AD score (Table 2).

**Figure 2:**
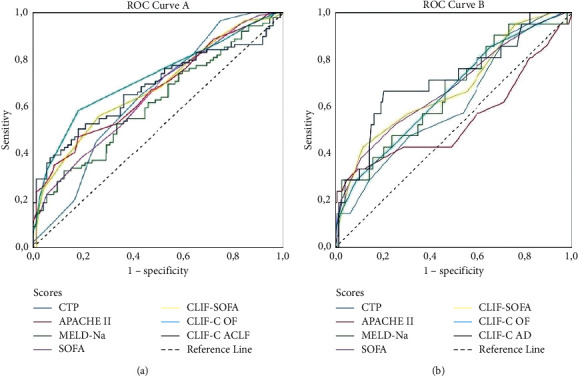
Comparison of the general and liver-specific prognostic scores to predict in-hospital mortality by AUROC in (a) patients with ACLF and (b) AD of cirrhosis. ACLF, acute-on-chronic liver failure; AD, acute decompensation; CLIF-C, Chronic Liver Failure Consortium; APACHE II, Acute Physiology and Chronic Health Evaluation II; MELD, Model for End-Stage Liver Disease; MELD-Na, sodium MELD; CTP, Child–Turcotte–Pugh; SOFA, Sequential Organ Failure Assessment; CLIF-SOFA, CLIF Sequential Organ Failure Assessment; CLIF-C OF, CLIF-C organ failure.

**Figure 3:**
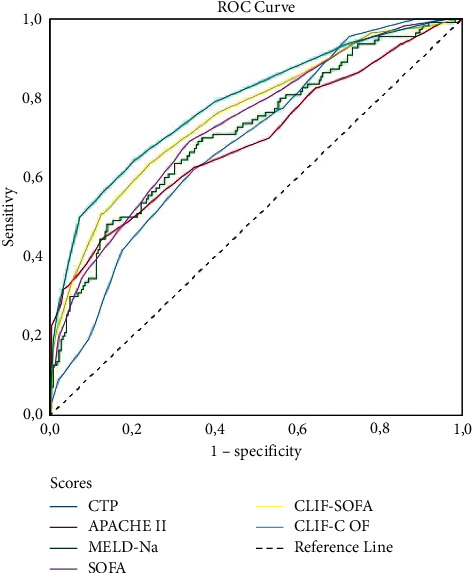
Comparison of general and liver-specific prognostic scores calculated on day 1 to predict in-hospital mortality by receiver operating characteristic curves in all patients either with ACLF or AD of cirrhosis. ACLF, acute-on-chronic liver failure; CLIF-C, Chronic Liver Failure Consortium; APACHE II, Acute Physiology and Chronic Health Evaluation II; MELD, Model for End-Stage Liver Disease; MELD-Na, sodium MELD; CTP, Child–Turcotte–Pugh; SOFA, Sequential Organ Failure Assessment; CLIF-SOFA, CLIF Sequential Organ Failure Assessment; CLIF-C OF, CLIF-C organ failure.

ROC curves were used to assess the ability of the scores calculated within 24 hours of admission to predict in-hospital mortality for all patients and those with either ACLF or AD (Figure 2 and Table 3). SOFA (0.753; 95% CI: 0.708–0.796), CLIF-SOFA (0.776, 95% CI: 0.724–0.827), and CLIF-C OF scores (0.807; 95% CI: 0.758–0.855) had the highest AUC values in all critically ill cirrhotic patients, and these scores were not statistically different from each other (*p*=0.083) (Figure 3). Since the CLIF-C OF score was considered the reference score (gold standard), the AUC values of the MELD (*p*=0.013), MELD-Na (*p*=0.037), and APACHE II (*p*=0.042) scores were significantly lower than CLIF-C OF.

In patients with ACLF, higher AUC values were obtained with CLIF-C OF (0.749; 95% CI: 0.679–0.820), when compared to CLIF-C ACLF (0.665; 95% CI: 0.585–0.745; *p*=0.029), SOFA (*p*=0.037), and MELD-Na (*p*=0.002), but no CLIF-SOFA score (*p*=0.085). It is of note that CLIF-SOFA, SOFA, and CLIF-C AD had the highest AUC values in those patients with AD of cirrhosis, with no statistical difference (*p*=0.971) (Figure 2 and Table 3).

**Figure 4:**
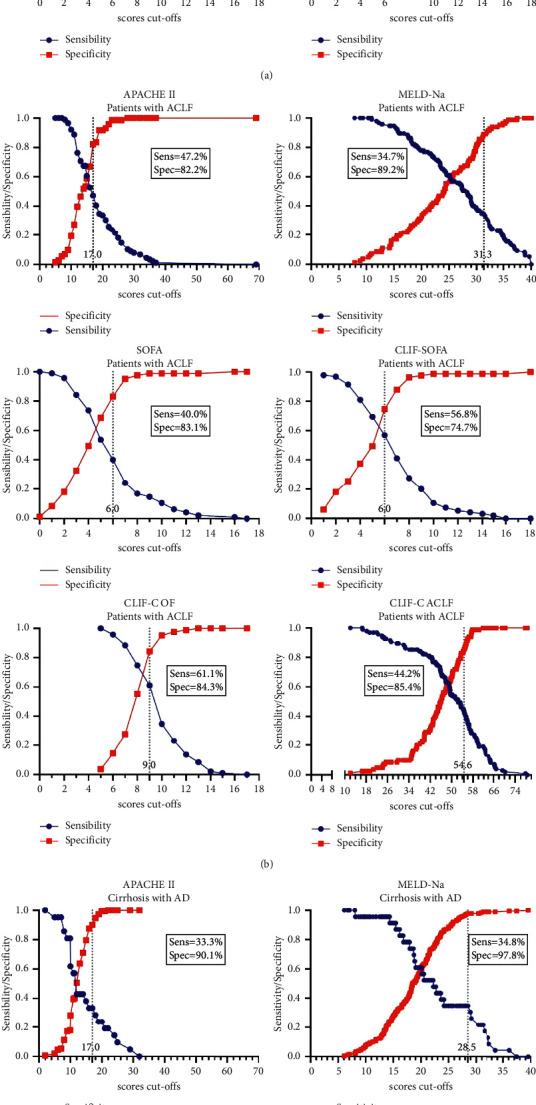
(a) Curves of sensibility and specificity relative to different cut-off values (TG-ROC curves) of the mortality prognostic scores and sensibility and specificity of the optimal cut-off point in 382 patients either with ACLF or AD of cirrhosis. (b) Curves of sensibility and specificity relative to different cut-off values (TG-ROC curves) of the mortality prognostic scores and sensibility and specificity of the optimal cut-off point in 178 patients with ACLF of cirrhosis. (c) Curves of sensibility and specificity relative to different cut-off values (TG-ROC curves) of the mortality prognostic scores and sensibility and specificity of the optimal cut-off point in 204 patients with AD of cirrhosis.

The most discriminative cut-off point was determined using the highest Youden Index (for each prognostic score), and corresponding sensitivity, specificity, PPV, NPV, LR +, and LR− are shown in Table 4 and Figures 4(a)–4(c).  Figure 4(a) shows that the most discriminative cut-off obtained to all patients was similar between patients with ACLF and AD to APACHE II score (cut-off = 17). But higher cut-off points considered optimal were observed for the other analyzed mortality prognostic scores in the group of patients with ACFL compared with patients with AD of cirrhosis (Figures 4(b) and 4(c)). Notably, the optimal cut-off points were associated with higher specificity values, reaching maximum values for MELD-Na (89.2% and 97.8%, respectively in patients with ACLF and AD of cirrhosis).

In Table 5, we observe that the quintiles of the cut-off points for the APACHE II, MELD-Na, SOFA, CLIF-SOFA, and CLIF-C OF scores produce a low prediction for death in patients with cirrhosis with AD, even considering the 80th percentile. More consistent values were observed in the group with ACLF.

## 4. Discussion

This 6-year retrospective single-center study evaluated 382 critically ill patients with cirrhosis with and without ACLF, which was assessed within 24 hours of ICU admission. In-hospital mortality rate was 31%, in accordance with previous reports, showing an increase in survival of cirrhotic patients admitted to the ICU in recent years [7, 14]. In the present study, mortality was associated with clinical and laboratory parameters, previously associated with prognosis, in several other studies, including bacterial infections or sepsis [8, 35, 37], HE [38], AKI [32, 39], and ACLF [26, 31] at admission; baseline sodium [12, 31, 40], creatinine [12, 31, 40], bilirubin [12, 31, 40], INR [12, 31, 40], and leukocyte counts [12, 31, 40]; and need for mechanical ventilation [12, 31] or vasopressors [12, 31].

We included several prognostic scores to predict mortality in cirrhotic patients since liver and ICU scores have been proposed. However, the discriminatory power of these scores in critically ill cirrhotic patients is not well defined. Few studies have analyzed the predictive value of MELD score, including its modified versions to predict in-hospital mortality [12].

As previously described [31, 33], occurrence and severity of ACLF, defined by either CLIF-C or NACSELD criteria, were robust predictors of hospital mortality. In the present cohort, 91% and 53% of the patients who had ACLF at admission, respectively, by NACSELD and CLIF-C definition, died at the hospital. Using AUC analysis, the authors have compared the performance of general and liver-specific scores in their ability to predict in-hospital mortality in all cirrhotic patients admitted to the ICU as well as in those patients with or without ACLF. We have found that SOFA, CLIF-SOFA, and CLIF-COF scores had a better performance when compared to other general ICU and liver-specific scores, such as APACHE II, CCI, MELD, and its variants and CTP scores in the entire group of patients. SOFA score was previously associated with better prognostication, particularly when compared to MELD and CTP scores, for every patient with cirrhosis in several [9, 12, 41–44] but not all publications [45]. Due to these findings, CLIF-C investigators adapted SOFA score to incorporate INR, instead of platelet count (CLIF-SOFA), to better evaluate liver dysfunction and organ failure (CLIF-C OF) in critically ill cirrhotic patients [26, 27, 31]. It is worth mentioning that both scores outperformed SOFA in their ability to predict mortality in the present study. Other authors have found similar performance of SOFA and CLIF-SOFA [14, 40, 46, 47] or better performance of CLIF-SOFA over SOFA [48] when calculated within 24 hours of ICU admission and even better prognostication when recalculated after 72 hours in some [14, 46] but not all studies [47]. Those differences may be due to comparison of heterogeneous cohorts comprised of patients from different genetic backgrounds and more importantly with differing percentages of organ failures. Another key point to better understand these discrepancies is to recognize that most of those studies, evaluating the accuracy of the prognostic scores, used distinct time intervals to assess outcomes, including in-ICU [25], in-hospital [40, 42, 45, 46], 28-day [49, 50], 6-week [9], and 90-day mortality [50]. Few studies have assessed accuracy of general ICU and liver-specific prognostic scores in critically ill patients with cirrhosis according to the presence of ACLF [16, 41, 51, 52], and none of them have compared the accuracy of those scores to predict in-hospital mortality. In the present study, CLIF-C OF outperformed other scores including CLIF-C ACLF and CLIF-SOFA in patients with ACLF and CLIF-C AD had a good accuracy to predict mortality in those patients with AD. Our findings were different from those reported by other North American and European authors [51, 52], who have reported better prognostication in patients with ACLF using CLIF-C ACLF score. It is important to mention that this score was prospectively developed using the CANONIC cohort of hospitalized Caucasian patients with cirrhosis, not particularly in the ICU, with external validation using an independent French cohort [27]. The CLIF-C ACLF score is calculated combining the CLIF-C OF score with age and leukocyte count and outperformed, up to now, all other prognostic scores in the evaluation of cirrhotic patients with ACLF. One possible reason for the discrepancies observed in the present study, in face of all others, is not just the short time interval to assess mortality (in-hospital mortality), but also some clinical features presented by our patients, such as older age, with mean age at least 10 years higher when compared to other studies, and a high index of comorbidities. Different genetic backgrounds could also be responsible since CLIF-C ACLF was validated almost exclusively in European and North American Caucasian patients [27, 51]. On the other hand, CLIF-C AD had better accuracy to predict mortality in comparison to CTP, MELD, and MELD-Na in our patients with AD of cirrhosis. CLIF-C AD was also developed by the CANONIC group of investigators [28], using the following parameters: age, INR, serum creatinine, and leukocyte count. Other [53–55], but not all [50], studies have disclosed similar findings. Our results report a good ability of CLIF-C AD to predict in-hospital mortality in cirrhotic patients admitted to the ICU with AD of cirrhosis, per definition without ACLF.

To increase the prognostic accuracy of the mentioned scores, several authors have suggested that incorporation of lactate in MELD [56] and CTP [25], as well as CLIF-C ACLF [52] scores, could increase their capacity of predicting mortality. One limitation of our study was the unavailability of data concerning lactate to confirm the findings.

It is noteworthy that in this study, we did not aim to assess the clinical course of patients with ACLF. Therefore, the scores were not recalculated throughout the hospital stay. These data can be evaluated in further studies. Additionally, the low rate of transplantation in this cohort helps to avoid a competing-risk analysis, since higher rates of transplantation during hospitalization could modify prognostic scores accuracy.

In conclusion, lower mortality rates are nowadays observed in cirrhotic patients admitted to the ICU, particularly in the absence of ACLF. In their ability to predict survival, for patients admitted in the ICU, the following scores outperformed other prognostic scores: CLIF-C OF and CLIF-SOFA, for all cirrhotic patients, CLIF-C OF, for patients with ACLF, and SOFA, CLIF-SOFA, and CLIF-C AD, for patients with AD. Stratification of patients with or without ACLF at admission, as well as during hospital stay, is important to improve prognostication. According to the clinical scenario, different scores should be used to provide prognosis to patients with cirrhosis in the ICU.

## Figures and Tables

**Table 1:** Demographics, clinical features, and outcomes of cirrhotic patients admitted to the ICU.

Characteristics	All patients (*n* = 382)	Patients with ACLF (*n* = 178)	Cirrhosis with AD (*n* = 204)
Age (years)	67.3 ± 10.6	58.2 ± 24.7	62.4 ± 20.8
Male sex	280 (73%)	133 (74.7%)	147 (72.1%)
Ascites	321 (84%)	154 (86.5%)	167 (81%)
Hepatic encephalopathy	211 (55%)	110 (61.7%)	101 (49.5%)
Variceal bleeding	24 (6%)	12 (6.7%)	12 (5.9%)
Bacterial infections/sepsis	233 (61%)	98 (55%)	135 (66%)
Acute kidney injury	123 (32%)	80 (45%)	43 (21%)
ACLF by CLIF-C criteria	178 (47%)		
(i) Grade I	90 (24%)		
(ii) Grade II	36 (9%)		
(iii) Grade III	52 (14%)		
ACLF by NACSELD criteria	33 (9%)		
Serum sodium (mEq/L)^1^	136 (132–140) 109–157	137 (131–140) 109–157	136 (132–140) 109–155
Serum creatinine (g/dl)^1^	1.0 (0.7–1.6) 0.3–8.4	1.4 (0.87–2.5) 0.4–8.4	0.9 (0.6–1.2) 0.3–3.5
Serum albumin (mg/dl)^1^	2.5 (2.2–2.9) 0.4–4.4	2.5 (2.1–2.9) 1.0–4.2	2.6 (2.3–2.9) 0.4–4.4
Serum bilirubin (mg/dl)^1^	2.0 (1.2–4.2) 0.4–36.7	2.3 (1.2–5.3) 0.4–36.7	2.0 (1.1–3.10) 0.4–27
INR^1^	1.7 (1.4–2.1) 0.51–13.44	1.87 (1.4–2.3)0.93–12.7	1.6 (1.4–2.0) 0.5–3.4
Leukocyte count (109/L)^1^	7,330 (5,060–11,030) 1,070–45,090	8,670 (6,460–13,722) 1,070–45,090	6,545 (4,360–6,545) 1,140–24,120
MELD-Na	22 ± 8		
SOFA	4 ± 3		
CLIF-SOFA	5 ± 3		
CLIF-C OF	8 ± 2		
CLIF-C ACLF	48 + 12		
CLIF-C AD	57 + 9		
ICU length of stay^1^	4.0 (2.0–9.0) 0.8–51.0	5 (2–10) 1.0–51.0	3.0 (1–6) 1.0–29.0
Hospital length of stay^1^	11.0 (8.0–18.0) 1.0–103.0	16.5 (9.0–12.5) 1.0–103.0	10 (7–16) 1.0–64.0
Mortality	118 (30.9%)	95 (53.4%)	23 (11.3%)

^1^Expressed by median (25th–75th)/min–max; ACLF, acute-on-chronic liver failure; CLIF-C, Chronic Liver Failure Consortium; NACSELD, North American Consortium for the Study of End-Stage Liver Disease; MELD, Model for End-Stage Liver Disease; SOFA, Sequential Organ Failure Assessment; OF, organ failure; AD, acute decompensation; ICU, intensive care unit.

**Table 2:** Comparison of survivors and nonsurvivors with cirrhosis admitted to the ICU.

Parameters	Survivors (*n* = 264)	Nonsurvivors (*n* = 118)	*p* values
*Age (years)*	67.6 + 10.4.5	66.7 + 11.3	0.88
*Male sex*	200 (76%)	80 (68%)	0.11
*Clinical features*			
Ascites	223 (85%)	98 (83%)	0.73
Bacterial infections/sepsis	130 (49%)	103 (87%)	<0.0001
Hepatic encephalopathy	128 (49%)	83 (70%)	<0.0001
Acute kidney injury	49 (19%)	70 (59%)	<0.0001
Variceal bleeding	13 (5%)	11 (9%)	0.11
AD of cirrhosis	181 (69%)	23 (20%)	<0.001
ACLF by CLIF criteria	83 (31%)	95 (80%)	< 0.001^1^
(i) Grade I	59 (22%)	31 (26%)	
(ii) Grade II	15 (6%)	21 (18%)	
(iii) Grade III	9 (3%)	43 (36%)	
ACLF by NACSELD criteria	3 (1%)	30 (25%)	<0.0001
*Laboratory features*			
Serum sodium (mEq/L)	135.2 + 7.2	135.2 + 8,1	0.11
Serum creatinine (mg/dl)	1.2 + 1.0	1.8 + 1.4	<0.0001
Serum bilirubin (mg/dl)	3.1 + 4.0	5.7 + 7.0	<0.0001
Serum albumin(g/dl)	2.6 + 0.5	2.5 + 0.5	0.84
INR	1.8 + 0.9	2.2 + 1.5	0.01
Leukocyte count (109/L)	7512 + 4105	11808 + 7321	<0.0001
Organ support admission			
Vasopressor therapy	4 (1, 5%)	13 (11%)	<0.0001
Mechanical ventilation	4 (2%)	24 (20%)	<0.0001
*Scores*			
CCI	6.5 + 3.4	7.8 + 2.9	0.008
Apache II	13.1 + 3.9	18.3 + 8.6	0.001
CTP	9.6 + 2.2	11.1 + 1.7	0.02
MELD	17.8 + 6.2	24.6 + 8.6	0.001
MELD-Na	20.2 + 6.5	26.5 + 7.9	0.001
iMELD	41.9 + 9.4	47.0 + 12.4	<0.0001
MESO index	1.3 + 0.5	1.8 + 0.7	<0.0001
SOFA	3.7 + 2.0	6.1 + 3.0	<0.0001
CLIF-SOFA	4.1 + 2.1	6.9 + 3.1	<0.0001
CLIF-C OF	7.2 + 1.7	9.7 + 2.3	<0.0001
CLIC-C ACLF	44.6 + 10.0	50.4 + 13.1	0.03
CLIF-C AD	56.6 + 9.2	63.2 + 9.3	0.48

^1^Chi-square for trend; AD, acute decompensation; ACLF, acute-on-chronic liver failure; CLIF-C, Chronic Liver Failure Consortium; NACSELD, North American Consortium for the Study of End-Stage Liver Disease; CCI, Charlson comorbidities index; APACHE II, Acute Physiology and Chronic Health Evaluation II; MELD, Model for End-Stage Liver Disease; MELD-Na, sodium MELD; MESO index, MELD to serum sodium ratio index; iMELD, integrated-MELD; CTP, Child–Turcotte–Pugh; SOFA, Sequential Organ Failure Assessment; CLIF-SOFA, CLIF Sequential Organ Failure Assessment; CLIF-C OF, CLIF-C organ failure; CLIF-C ACLF, CLIF acute-on-chronic liver failure; CLIF-C AD, CLIF-C acute decompensation; ICU, intensive care unit.

**Table 3:** Performance of prognostic scores in cirrhotic patients admitted to the ICU.

Score	AUC	Standard error	95% confidence interval^1^	*p* values
*All patients* (*n* = 382)				
CTP	0.701	0.027	0.648–0.754	<0.001
APACHE II	0.695	0.032	0.632–0.759	<0.001
MELD	0.727	0.030	0.669–0.785	<0.001
MELD-Na	0.729	0.029	0.670–0.784	<0.001
MESO index	0.723	0.030	0.665–0.781	<0.001
iMELD	0.640	0.033	0.576–0.705	<0.001
SOFA	0.753	0.027	0.708–0.796	<0.001
CLIF-SOFA	0.776	0.0269	0.724–0.827	<0.001
CLIF-C OF	0.807	0.025	0.758–0.855	<0.001
CCI	0.627	0.029	0.571–0.683	<0.001
*Patients with ACLF* (*n* = 178)				
CTP	0.662	0.041	0.581–0.743	0.002
APACHE II	0.674	0.042	0.592–0.755	<0.001
MELD	0.647	0.041	0.566–0.729	0.01
MELD-Na	0.638	0.041	0.557–0.719	0.01
MESO index	0.633	0.041	0.552–0.714	0.02
iMELD	0.540	0.043	0.455–0.625	0.27
SOFA	0.677	0.039	0.600–0.754	0.001
CLIF-SOFA	0.698	0.039	0.622–0.773	<0.001
CLIF-C OF	0.749	0.036	0.679–0.820	<0.001
CLIF-C ACLF	0.665	0.041	0.585–0.745	<0.001
*Patients with AD of cirrhosis* (*n* = 204)				
CTP	0.650	0.060	0.532–0.768	0.10
APACHE II	0.543	0.083	0.380–0.707	0.52
MELD	0.650	0.072	0.510–0.790	0.06
MELD-Na	0.676	0.065	0.549–0.803	0.02
MESO index	0.654	0.070	0.519–0.790	0.04
iMELD	0.682	0.077	0.532–0.832	0.02
SOFA	0.715	0.063	0.592–0.837	0.008
CLIF-SOFA	0.716	0.062	0.595–0.837	0.008
CLIF-C AD	0.695	0.065	0.569–0.822	0.001

^1^Asymptotic normal 95% CI; AD, acute decompensation; ACLF, acute-on-chronic liver failure; CLIF-C, Chronic Liver Failure Consortium; NACSELD, North American Consortium for the Study of End-Stage Liver Disease; CCI, Charlson comorbidities index; APACHE II, Acute Physiology and Chronic Health Evaluation II; MELD, Model for End-Stage Liver Disease; MELD-Na, sodium MELD; MESO index, MELD to serum sodium ratio index; iMELD, integrated-MELD; CTP, Child–Turcotte–Pugh; SOFA, Sequential Organ Failure Assessment; CLIF-SOFA, CLIF Sequential Organ Failure Assessment; CLIF-C OF, CLIF-C organ failure; CLIF-C ACLF, CLIF acute-on-chronic liver failure; CLIF-C AD, CLIF-C acute decompensation.

**Table 4:** Performance of different prognostic scores in predicting mortality using the optimal cut-off point in all patients, patients with ACLF (CLIF-C ACLF) and patients with AD of cirrhosis (CLIF-C AD).

Score	Cut-off point	Youden index	Sens^1^ (%)	Spec^1^ (%)	PPV^1^	NPV^1^	LR+	LR−
APACHE II	17	0.321	44.6 (35.6–56.9)	87.6 (82.6–91.3)	63.6 (52.5–73.5)	76.4 (70.8–81.1)	3.6	0.63
CTP	10	0.296	64.4 (55.4–72.5)	65.2 (59.2–70.6)	45.2 (37.9–52.8)	80.4 (74.5–85.1)	1.9	0.55
SOFA	4	0.378	70.3 (61.6–72.8)	67.4 (61.6–72.8)	49.1 (41.7–56.7)	83.6 (78.0–87.9)	2.2	0.44
CLIF-SOFA	5	0.414	65.3 (56.3–73.2)	74.1 (70.6–80.9)	55.0 (46.7–63.0)	83.1 (77.8–87.3)	2.7	0.46
CLIF-C OF	8	0.469	67.0 (58.1–74.8)	79.9 (74.7–84.3)	59.9 (51.3–67.8)	84.4 (79.4–88.4)	3.3	0.41
MELD	23.2	0.379	65.3 (56.3–73.2)	76.1 (70.6–80.9)	55.0 (46.-63.0)	83.1 (77.8–87.3)	2.7	0.46
MELD-Na	27.2	0.367	50.9 (41.9–59.7)	85.8 (81.1–89.5)	61.9 (51.9–70.9)	79.4 (74.3–83.7)	3.6	0.57
iMELD	47	0.296	52.5 (43.6–61.3)	77.0 (71.5–81.7)	50.8 (42.1–59.5)	78.2 (72.8–82.8)	2.3	0.62
MESO index	1.8	0.404	54.2 (45.3–63.0)	85.1 (80.2–88.9)	62.1 (52.5–70.9)	80.4 (75,4–84.7)	3.6	0.54
CCI	4	0.259	89.7 (82.9–94.0)	36.1 (30.6–42.1)	38.5 (32.9–44.4)	88.8 (81.4–93.5)	1.4	0.28
CLIF-C ACLF^2^	54.6	0.296	44.2 (34.6–54.2)	84.2 (74.7–90.5)	76.4 (63.7–85.6)	56.6 (47.7–65.0)	2.8	0.66
CLIF-C AD^3^	62.9	0.394	60.9 (40.8–77.8)	78.0 (71.3–81.4)	26.4 (16.4–39.6)	93.9 (88.8–96.8)	2.8	0.50

^1^Data expressed in % and 95% CI; ^2^only patients with ACLF (*N* = 178); ^3^only patients with AD of cirrhosis (*N* = 204). Sens: sensitivity; Spec: specificity; PPV: positive predictive value; NPV: negative predictive value; LR+: likelihood ratio positive; LR−: likelihood ratio negative.

**Table 5:** Cut-off points, sensibility, specificity, area under curve, and positive predictive value (to predict mortality) of the quintiles of APACHE II, MELD-Na, SOFA, CLIF-SOFA, CLIF-C ACLF, and CLIF-C AD scores.

Score	20^th^					40^th^					60^th^					80^th^				
	Co	Se	Sp	AUC	PPV	Co	Se	Sp	AUC	PPV	Co	Se	Sp	AUC	PPV	Co	Se	Sp	AUC	PPV
*All patients* (*N* = 382)																				
APACHE II	10	86.4	25.3	0.558	36.1	12	70.0	47.1	0.586	39.3	15	56.4	72.9	0.646	50.4	18	38.2	91.1	.646	67.7
MELD-Na	15.4	93.2	25.7	0.594	36.2	19.5	76.3	47.5	0.619	39.6	23.4	63.6	70.5	0.670	49.3	29.0	40.7	89.7	0.652	64.0
SOFA	2	94.1	26,9	0.605	36.5	4	70.3	67.4	0.689	49.1	5	51,7	83,8	0.675	58.1	6	37.3	93.2	0.652	71.0
CLIF-SOFA	3	87.3	39.0	0.632	39.0	4	77.1	60.2	0.687	46.4	5	65.3	76.1	0.707	55.0	7	36.4	95.5	0.659	78.2
CLIF-C OF	6	94.1	29.9	0.620	37.5	7	80.5	60.2	0.704	47.5	8	66.9	79.9	0.734	59.8	10	30.5	98.1	0.643	87.8
*Patients with ACFL* (*N* = 178)																				
APACHE II	12	76.4	39.7	0.581	60.7	14	67.4	52.1	0.597	63.2	17	47.2	82.2	0.647	76.4	21.4	30.3	93.2	0.617	84.4
MELD-Na	18.1	85.3	25.3	0.553	56.6	23.7	66.3	47.0	0.567	58.9	28.4	47.4	68.7	0.580	63.4	32.3	29.5	91.6	0.605	80.0
SOFA	3	84.2	32.5	0.584	58.5	5	53.7	68.7	0.612	66.2	6	40.0	83.1	0.616	73.1	7	24.2	95.2	0.597	85.2
CLIF-SOFA	4	81.1	37.3	0.592	59.7	6	56.8	74.7	0.658	72.0	7	41.1	88.0	0.645	79.6	8	27.4	96.4	0.619	89.7
CLIF-C OF	8	74.7	55.4	0.651	65.7	9	61.1	84.3	0.727	81.7	9.4	61.1	84.3	0.727	81.7	11	23.2	97.6	0.604	91.7
CLIF-CACLF	39.2	83.2	23.2	0.532	55.6	46.2	68.4	51.2	0.598	61.9	52.0	51.6	73.2	0.624	69.0	56.9	31.6	93.9	0.627	85.7
*Patients with AD of cirrhosis* (*N* = 204)																				
APACHE II	10	61.9	28.3	0.451	10.7	11.2	57.1	39.5	0.483	11.5	13	42.9	63.8	0.533	14.1	16	33.3	87.5	0.604	26.9
MELD-Na	14.1	95.7	21.9	0.588	13.7	17.9	78.3	42.1	0.602	14.9	20.3	56.5	62.4	0.594	16.3	23.6	43.5	83.1	0.633	25.0
SOFA	2	87.0	30.9	0.589	13.8	3	69.6	54.1	0.619	16.2	4	56.5	75.7	0.661	22.8	5	43.5	90.1	0.668	35.7
CLIF-SOFA	2	95.7	26.0	0.608	14.1	3	69.6	45.3	0.574	13.9	4	60.9	70.7	0.658	20.9	5	47.8	88.4	0.681	34.4
CLIF-C OF	6	87.0	37.0	0.620	14.9	7	47.8	75.1	0.615	19.6	7	47.8	75.1	0.615	19.6	8	34.8	91.2	0.630	33.3
CLIF-CAD	50	87.0	21.5	0.542	12.6	54.4	73.9	41.8	0.579	14.2	58	65.2	63.8	0.645	19.0	64.9	52.2	84.2	0.682	30.0

Co = cut-off; Se=sensibility; Sp = specificity; AUC = area under curve; PPV = positive predictive value.

## Abbreviations

AD: Acute decompensation
ACLF: Acute-on-chronic liver failure
CLIF-C: Chronic Liver Failure Consortium
NACSELD: North American Consortium for the Study of End-Stage Liver Disease
CCI: Charlson comorbidities index
APACHE II: Acute Physiology and Chronic Health Evaluation II
MELD: Model for End-Stage Liver Disease
MELD-Na: Sodium MELD
MESO index: MELD to serum sodium ratio index
iMELD: Integrated-MELD
CTP: Child–Turcotte–Pugh
SOFA: Sequential Organ Failure Assessment
CLIF-SOFA: CLIF Sequential Organ Failure Assessment
CLIF-C, OFCLIF-C: Organ failure
CLIF-C ACLF: CLIF acute-on-chronic liver failure
CLIF-C AD, CLIF-C: Acute decompensation.
